# A new genomic blueprint of the human gut microbiota

**DOI:** 10.1038/s41586-019-0965-1

**Published:** 2019-02-11

**Authors:** Alexandre Almeida, Alex L. Mitchell, Miguel Boland, Samuel C. Forster, Gregory B. Gloor, Aleksandra Tarkowska, Trevor D. Lawley, Robert D. Finn

**Affiliations:** 1 European Bioinformatics Institute (EMBL-EBI), Wellcome Genome Campus, Hinxton, UK; 2 Wellcome Sanger Institute, Wellcome Genome Campus, Hinxton, UK; 3 Centre for Innate Immunity and Infectious Diseases, Hudson Institute of Medical Research, Clayton, Victoria Australia; 4 Department of Molecular and Translational Sciences, Monash University, Clayton, Victoria Australia; 5 Department of Biochemistry, University of Western Ontario, London, Ontario Canada

**Keywords:** Microbiome, Metagenomics, Bacterial genomics, Metagenomics

## Abstract

The composition of the human gut microbiota is linked to health and disease, but knowledge of individual microbial species is needed to decipher their biological roles. Despite extensive culturing and sequencing efforts, the complete bacterial repertoire of the human gut microbiota remains undefined. Here we identify 1,952 uncultured candidate bacterial species by reconstructing 92,143 metagenome-assembled genomes from 11,850 human gut microbiomes. These uncultured genomes substantially expand the known species repertoire of the collective human gut microbiota, with a 281% increase in phylogenetic diversity. Although the newly identified species are less prevalent in well-studied populations compared to reference isolate genomes, they improve classification of understudied African and South American samples by more than 200%. These candidate species encode hundreds of newly identified biosynthetic gene clusters and possess a distinctive functional capacity that might explain their elusive nature. Our work expands the known diversity of uncultured gut bacteria, which provides unprecedented resolution for taxonomic and functional characterization of the intestinal microbiota.

## Abstract (web summary)

The known species repertoire of the collective human gut microbiota is substantially expanded with the discovery of 1,952 uncultured bacterial species that greatly improve classification of understudied African and South American samples.

## Main

For the past decade, studies of the human gut microbiota have shown that the interplay between microbes and host is associated with various phenotypes of medical importance^1,2^. Shotgun metagenomic analysis methods can infer both taxonomic and functional information from complex microbial communities, guiding phenotypic studies aimed at understanding their potential roles in human health and disease. However, various strategies used for analysis of metagenomic datasets rely on high-quality reference databases^3^. This highlights the need for extensive and well-characterized collections of reference genomes, such as those from the Human Microbiome Project (HMP)^4,5^ and the Human Gastrointestinal Bacteria Genome Collection (HGG)^6–8^. Despite a new wave of culturing efforts, there is still a substantial but undetermined degree of unclassified microbial diversity within the gut ecosystem^6,8–11^. Whereas these unknown community members may have eluded current culturing strategies for a variety of reasons (for example, owing to lack of nutrients in growth media or their low abundance in the gut), they are likely to perform important biological roles that remain undiscovered. Thus, having access to a comprehensive catalogue of representative genomes and isolates from the intestinal microbiota is essential to gain new mechanistic insights.

Culture-independent and reference-free approaches have proved to be successful strategies for species discovery and characterization^12–16^. The most common approach is to perform de novo assembly of shotgun metagenomic reads into contig sequences and place them into different bins on the basis of sequence coverage and tetranucleotide frequency^15^—a process that enables the recovery of potential genomes, termed metagenome-assembled genomes (MAGs). Several studies have applied these methods to reconstruct large numbers of MAGs^13,17–19^, one of the most prominent being the recovery of thousands of genomes revealing new insights into the tree of life^16^.

Here we generated and classified a set of 92,143 MAGs from 11,850 human gut metagenome assemblies to expand our understanding of gut-associated microbiome diversity. We discovered 1,952 uncultured bacterial species and investigated their association with specific geographical backgrounds, as well as their unique functional capacity. This enabled new insights into which species and functions within this uncharacterized bacterial community might have underappreciated roles in the human gut environment.

## Large-scale discovery of uncultured species

**Extended Data Fig. 1:**
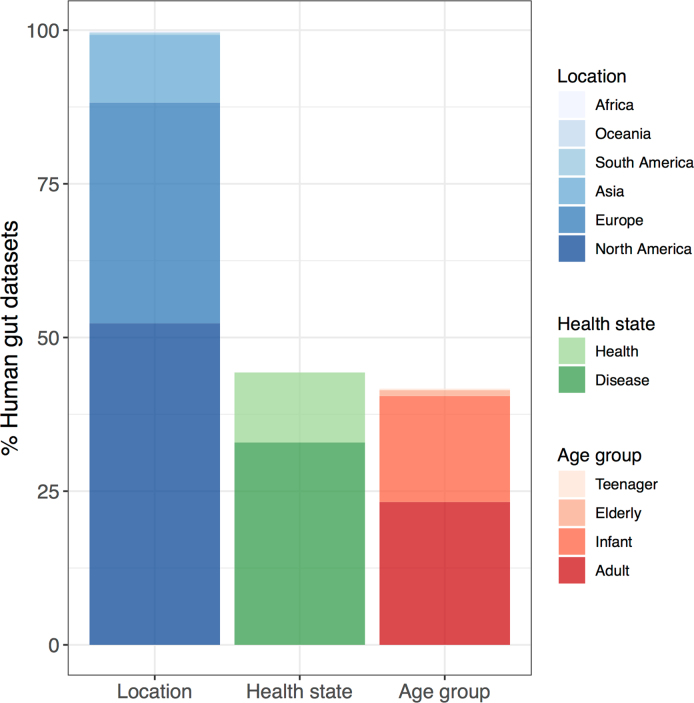
Metadata of the human gut datasets. Percentage of the 13,133 metagenomic datasets according to location, health state and age group of the individual sampled, as depicted in the figure key.

To perform a comprehensive characterization of the human gastrointestinal microbiota, we retrieved 13,133 human gut metagenomic datasets from 75 different studies (Supplementary Table 1 and Extended Data Fig. 1). Samples were collected mainly from North America (*n* = 6,869, 52%) or Europe (*n* = 4,716, 36%), reflecting a geographical bias in current human gut microbiome studies. The majority of datasets with available metadata were from diseased patients (*n* = 4,323, 33%) and adults (*n* = 3,053, 23%).

**Extended Data Fig. 2:**
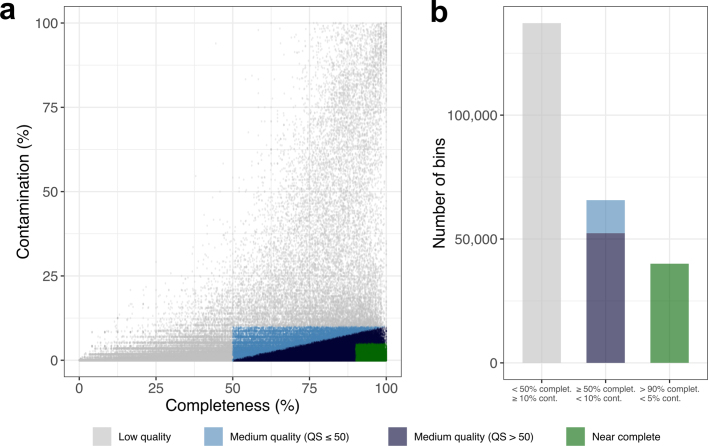
CheckM quality assessment of bins. **a**, Quality metrics estimated by CheckM for the 242,836 bins generated by MetaBAT. **b**, Number of bins recovered according to the level of genome completeness and contamination. QS = completeness – (5 × contamination). Source Data

**Extended Data Fig. 3:**
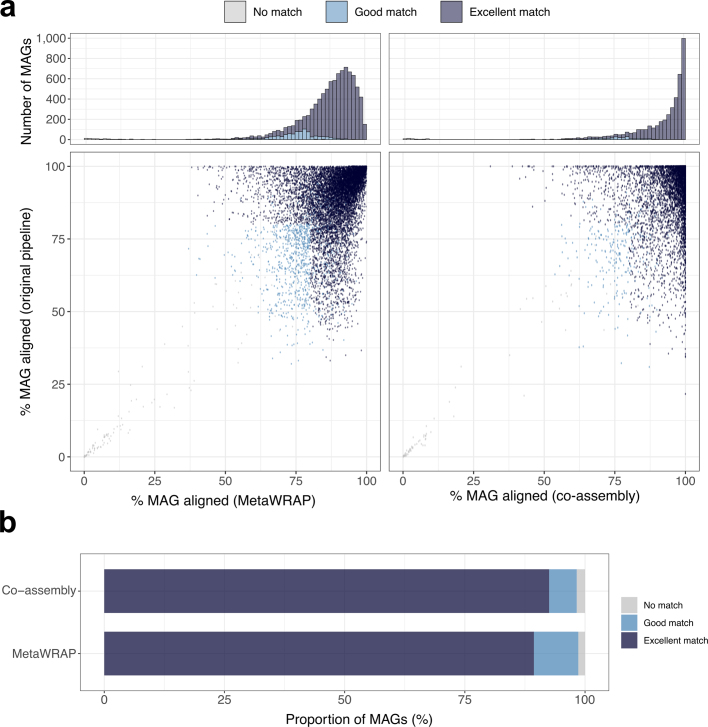
Technical reproducibility of MAGs. **a**, MAGs resulting from the MetaWRAP pipeline (left, *n* = 9,552) and from a modified co-assembly approach (right, *n* = 4,404) compared to the original MAGs generated with SPAdes and MetaBAT for 1,000 random datasets. A good match was defined as ≥95% ANI over ≥60% of alignment fraction, whereas an excellent match indicates ≥98% ANI over ≥80% alignment. **b**, Proportion of MAGs generated with each pipeline (MetaWRAP and co-assembly) coloured by their level of match to the original set.

Following assembly with SPAdes^20,21^, 11,850 of the 13,133 metagenome assemblies produced contigs that could undergo genomic binning by MetaBAT^15^, generating a total of 242,836 bins. The quality of each bin was evaluated with CheckM^22^ according to the level of genome completeness and contamination (Extended Data Fig. 2). On the basis of these metrics, 40,029 MAGs with more than 90% completeness and less than 5% contamination were obtained (hereafter referred to as ‘near-complete’^16^). We also generated 65,671 medium-quality^23^ MAGs (at least 50% completeness and less than 10% contamination), 52,347 of which had a quality score^16^ (QS) above 50 (defined as completeness – (5 × contamination)). The robustness of our MAGs was evaluated with two independent assembly/binning methodologies^24,25^ (see Supplementary Discussion and Extended Data Fig. 3), which showed the MAGs to be highly reproducible, independent of the method used for assembly or binning.

**Fig. 1:**
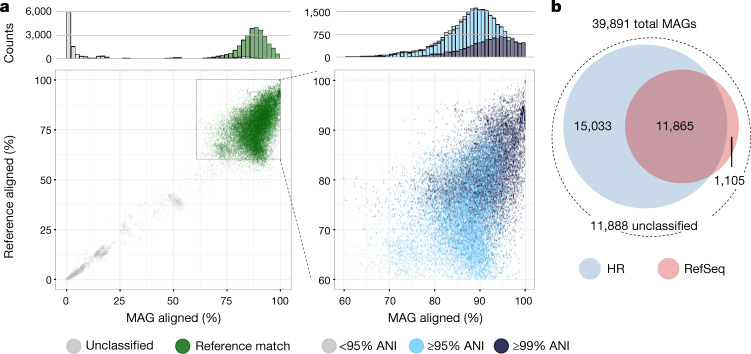
Thousands of metagenome-assembled genomes do not match isolate genomes. **a**, Left, near-complete (>90% completeness, <5% contamination) MAGs that matched the HR database (green; ≥95% average nucleotide identity over at least 60% of the genome) and those that could not be classified (grey). Right, expanded view of MAGs with an alignment fraction of at least 60%, coloured on the basis of the ANI in relation to the best matching HR genome. **b**, Number of near-complete MAGs matching HR (blue) and RefSeq (pink) alongside those that did not match any reference genome from either database.

**Extended Data Fig. 4:**
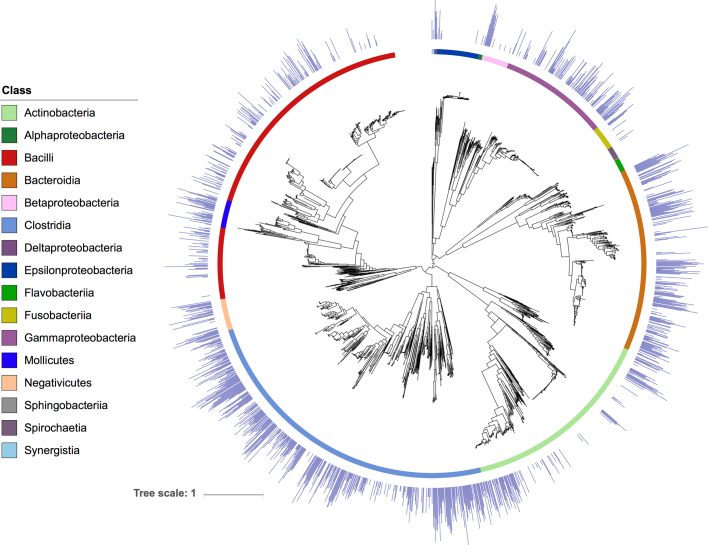
Phylogenetic diversity of the human-specific isolate genomes. Phylogenetic tree of the 2,468 HR genomes, labelled according to class, with the bar graphs in the outer layer depicting the log-transformed number of near-complete MAGs matching that corresponding genome.

As CheckM is unable to evaluate non-prokaryotic genomes, we investigated separately how many of our bins represented known eukaryotes or viral sequences (see Supplementary Discussion and Supplementary Table 2). However, for the main set of analyses, we focused on the 39,891 near-complete MAGs that CheckM resolved to bacterial lineages (Supplementary Table 3), excluding the remaining 139 MAGs that were assigned to the archaeal domain. To determine how many of the MAGs belong to species that have been isolated from pure bacterial cultures (that is, isolate genomes), we attempted to assign each MAG to a human-specific reference (HR) database, composed of 2,468 isolate genomes combined from the HMP catalogue and the HGG^8^ (Fig. 1). This dataset consisted of 956 individual species (553 specifically cultured from the gastrointestinal tract), defined according to previously reported genome thresholds for species delineation^26,27^ (at least 95% average nucleotide identity over at least 60% of the genome). In order to broaden the classification potential, we also compared the MAGs to the 8,778 complete bacterial genomes in RefSeq (Fig. 1b). Of the 39,891 MAGs, we were able to assign 26,898 to the HR dataset, and 12,970 to RefSeq, using a criterion of at least 60% of the MAG aligned with at least 95% average nucleotide identity (ANI). There was good coverage across different taxonomic groups within HR (Extended Data Fig. 4), with the three most frequent genomes assigned to the species *Ruminococcus bromii* (*n* = 1,255), *Alistipes putredinis* (*n* = 1,142) and *Eubacterium rectale* (*n* = 839). All are known colonizers of the human gut^28^, confirming that these species are common members of the intestinal microbiota.

**Extended Data Fig. 5:**
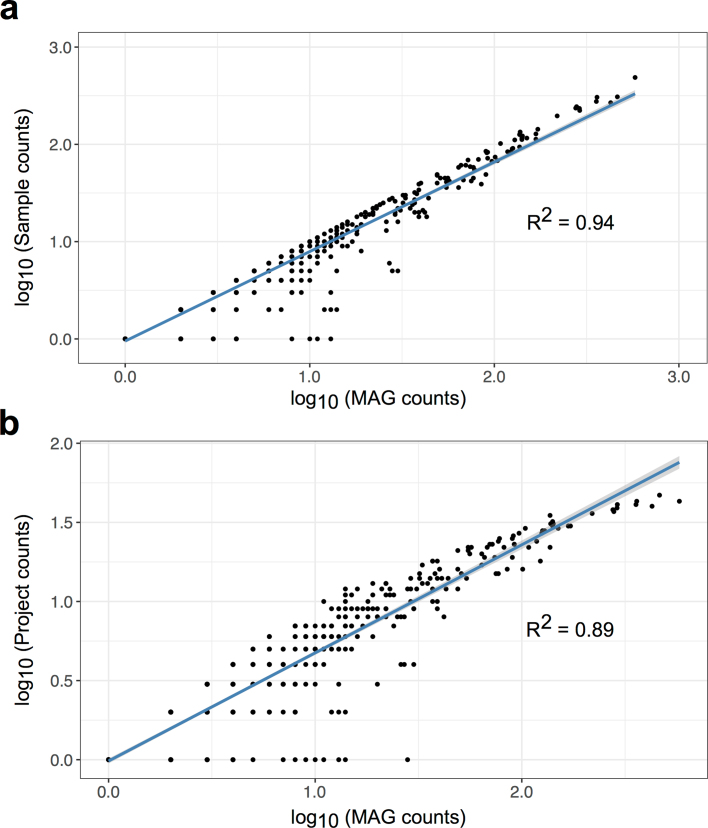
Analysis of Mash similarity clusters. Pearson correlation between the log-transformed number of MAGs and the corresponding number of distinct samples (**a**) or studies (**b**) per Mash cluster. Data points represent each of the 702 similarity groups (defined with a Mash distance <0.2). The coefficient of determination (*R*^2^) is depicted in each graph. Source Data

We subsequently focused on the 11,888 near-complete bacterial MAGs (30%) that were not assigned to HR or RefSeq (Fig. 1b). MAGs were dereplicated at an estimated species level (see Supplementary Discussion and Extended Data Fig. 5), yielding a total of 1,175 near-complete metagenomic species (MGS) with a median completeness of 96.5% (interquartile range (IQR) = 93.8–98.4%) and contamination of 0.8% (IQR = 0.0–1.5%) as estimated by CheckM.

**Extended Data Fig. 6:**
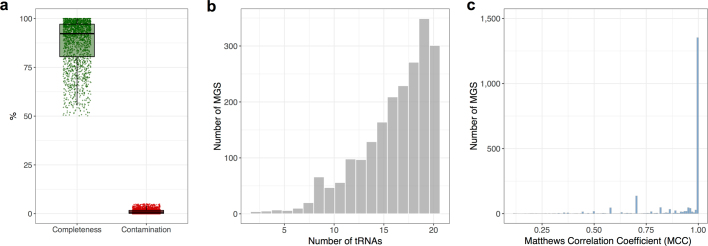
Quality metrics of the metagenomic species. **a**, Distribution of completeness (minimum: 55.5; Q1: 80.5; median: 92.3; Q3: 97.1; maximum: 100) and contamination levels (minimum: 0; Q1: 0.1; median: 0.8; Q3: 1.7; maximum: 4.1) estimated by CheckM for the 2,068 metagenomic species (MGS). **b**, Number of tRNAs coding for the 20 standard amino acids detected across the MGS genomes. **c**, MCC calculated for all the 2,068 MGS, based on the Mash clustering structure and an average amino acid identity threshold of 97%.

With this dataset of 1,175 MGS, we assessed how much of our original collection of human gut MAGs still remained unassigned by extending the analysis to both near-complete and medium-quality bacterial MAGs with a QS above 50 (*n* = 92,143, Extended Data Fig. 2). This resulted in identification of an additional 893 bacterial species with medians of 77.8% completeness (IQR = 68.9–85.8%) and 1.1% CheckM contamination (IQR = 0.2–2.0%), hereafter referred to as medium-quality MGS. Therefore, together with the 1,175 near-complete MGS, our analysis uncovered a total of 2,068 MGS (Extended Data Fig. 6), representing good-quality bacterial genomes absent from human-specific and high-quality reference databases (see Supplementary Discussion for further details on MAG quality assessment).

## Species characterization and distribution

**Fig. 2:**
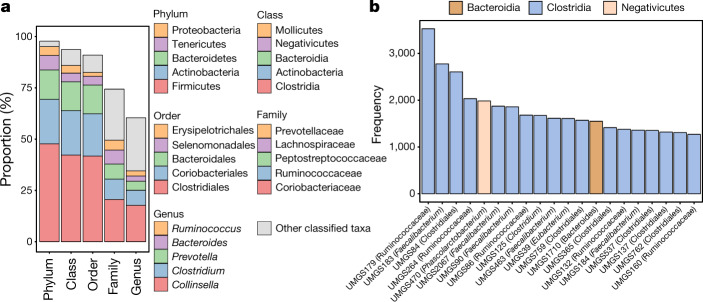
Taxonomy of the most prevalent uncultured gut bacterial species. **a**, Taxonomic composition of the 1,952 UMGS, with ranks ordered from top to bottom by their increasing proportion among the UMGS collection. Only the five most frequently observed taxa are shown in the legend, with the remaining lineages grouped as ‘other classified taxa’. **b**, Top 20 most prevalent UMGS genomes across the 13,133 metagenomic datasets, inferred from the level of genome coverage, read depth and evenness. Each species is coloured according to class, with the predicted taxon indicated in brackets.

Having identified 2,068 MGS in the human gut, we sought to determine their taxonomic classification and extend the analysis to more comprehensive reference databases. By complementing the phylogenetic inference method of CheckM with protein searches against the UniProt Knowledgebase (UniProtKB)^29^, we attempted to assign the most likely taxonomic lineage to each MGS. This approach, which utilizes both multiple marker genes and protein-level matches, is similar to those used by various analysis tools^30–32^ and provides a more reliable method for taxonomic assignment compared to traditional single-marker gene classifications (for example, based on the 16S rRNA gene). Using a species-level threshold^26,33^ (at least 60% of the proteins with at least 96% amino acid identity), we found that 94% of the MGS (*n* = 1,952) did not match any isolate genome within UniProtKB, and therefore represent uncultured candidate species. Of these 1,952 unclassified MGS (UMGS), 74% correspond to entirely ‘novel’ genomes as of August 2018 (see Supplementary Discussion and Supplementary Table 4). We were able to assign 98% and 94% of the UMGS at the phylum and class levels, respectively, and 91% to a known order (Fig. 2a). Interestingly, 26% of the UMGS were unassigned at the family level, while almost half (40%) could not be classified to a known genus, meaning that a substantial portion of the UMGS may belong to new families and/or genera. The three most frequently assigned families were Coriobacteriaceae (20.6%), Ruminococcaceae (9.9%) and Peptostreptococcaceae (7.4%), whereas the top genera were *Collinsella* (17.7%), *Clostridium* (7.3%) and *Prevotella* (4.4%). These data suggest that despite being known colonizers of the intestinal microbiota, these clades still contain considerable uncultured diversity. The *Clostridium* genus has been acknowledged as highly polyphyletic, with recent phylogenetic estimates suggesting that this group may span 121 genera belonging to 29 families^34^. Therefore, the detection of many uncultured species assigned to this genus may reflect current taxonomic limitations rather than a biological signal.

**Extended Data Fig. 7:**
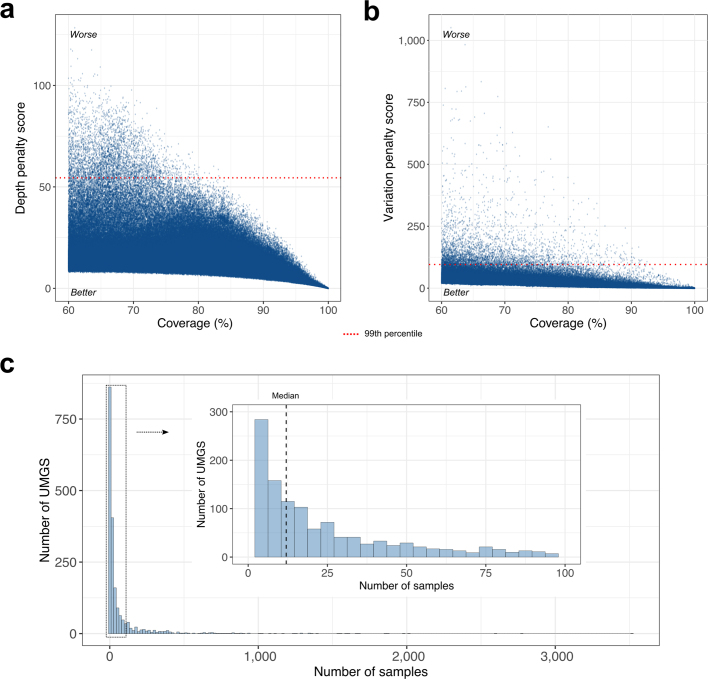
Defining genome presence and prevalence distribution. **a**, **b**, Depth (**a**) and variation (**b**) penalty scores plotted against the level of genome coverage of the 1,952 UMGS across all 13,133 metagenomic samples. The depth penalty score was calculated by multiplying the missing coverage (100 − genome coverage) by the log-transformed mean read depth. The variation penalty score was based on the missing coverage multiplied by the depth coefficient of variation (standard deviation of read depth divided by the mean). Dashed red lines correspond to the 99th percentile, set as the upper threshold used to define genome presence. **c**, Number of UMGS detected in the corresponding number of metagenomic samples. The distribution of UMGS found in up to 100 samples is illustrated as an inset. The vertical dashed line represents the median value of all data.

In order to determine the prevalence and abundance of the uncultured candidate species within each gut microbiome, we compared the raw reads from the original 13,133 metagenomic datasets to the UMGS collection. Prevalence was estimated by how many samples each genome was found in by taking into account the level of genome coverage, mean read depth and evenness (Extended Data Fig. 7). Half of the UMGS were found in at least 12 metagenomic samples (Extended Data Fig. 7c). The most frequently observed UMGS belong to the family Ruminococcaceae and the *Faecalibacterium* genus, and include mostly members from the Clostridia class (Fig. 2b).

**Fig. 3:**
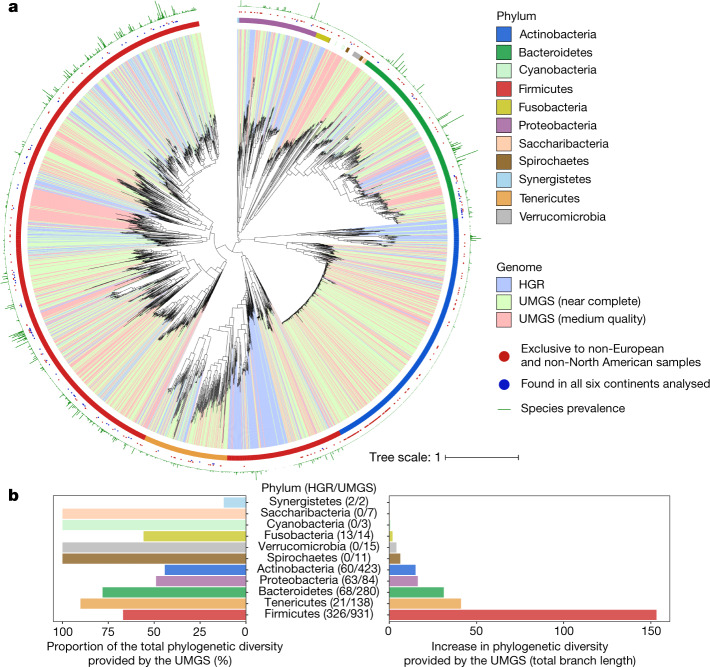
Phylogeny of reference and uncultured human gut bacterial genomes. **a**, Maximum-likelihood phylogenetic tree comprising the 553 genomes belonging to the HGR, and 1,952 to UMGS. Clades are labelled according to genome type (HGR, near-complete or medium-quality UMGS) and the corresponding phylum is depicted in the first outer layer. Blue and red dots in the second layer denote genomes that were found in at least one sample from all six continents analysed (Africa, Asia, Europe, North America, South America and Oceania), or exclusively detected in non-European, non-North American samples, respectively. Green bars in the outermost layer represent the prevalence of the genome among the 13,133 metagenomic datasets. **b**, Level of increase in phylogenetic diversity provided by the UMGS, relative to the complete diversity per phylum (left) and represented as absolute total branch lengths (right). The number of HGR and UMGS genomes assigned to each phylum is depicted in brackets (HGR/UMGS).

To place these uncultured species in context with the known bacterial colonizers of the human gut, we then positioned the UMGS within the gut-specific species from the HR database, hereafter referred to as the human gut reference (HGR). A maximum-likelihood phylogeny of the 1,952 UMGS and the 553 HGR genomes was built on the basis of the 40 marker genes extracted with specI^32^ (Fig. 3a). Phylogenetic analysis showed that the UMGS genomes expand the known diversity of the human gut bacterial lineages by 281%, on the basis of total branch lengths, with the largest increase within the Firmicutes phylum (Fig. 3b). Several uncultured genomes showing high phylogenetic similarity were retrieved belonging to Actinobacteria, particularly the *Collinsella* genus. This suggests that the genome-based boundaries between species and genus within this group are more tenuous compared to other human gut bacterial clades. Of note is that the UMGS included genomes belonging to Cyanobacteria (Gastranaerophilales), Saccharibacteria, Spirochaetes and Verrucomicrobia. These are likely to correspond to rarer or more difficult-to-culture clades from the human gut, as none had a representative isolate genome in the HGR database.

**Fig. 4:**
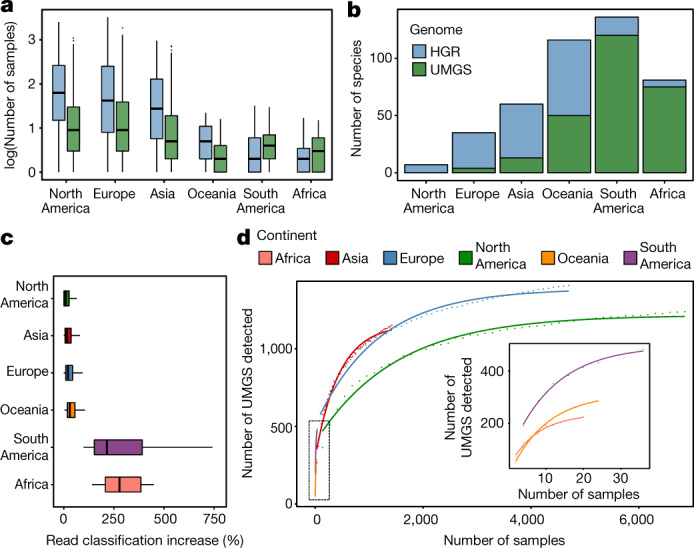
Geographical distribution of the samples and uncultured species. **a**, Distribution of the number of samples (log-transformed) that each HGR or UMGS present in at least one sample was found at a relative abundance above 0.01%. HGR genomes: *n* = 31 (Africa), *n* = 340 (Asia), *n* = 351 (Europe), *n* = 362 (North America), *n* = 86 (South America) and *n* = 129 (Oceania). UMGS genomes: *n* = 230 (Africa), *n* = 1,157 (Asia), *n* = 1,410 (Europe), *n* = 1,238 (North America), *n* = 482 (South America) and *n* = 287 (Oceania). **b**, Number of species found (abundance > 0.01%) in more than 20% of the samples from each geographical region. **c**, Percentage increase of the proportion of reads, partitioned by sample geographical location (Africa, *n* = 21; Asia, *n* = 1,447; Europe, *n* = 4,716; North America, *n* = 6,869; South America, *n* = 36; Oceania, *n* = 24), that were assigned to the HR, RefSeq and UMGS, in relation to HR and RefSeq alone. **d**, Accumulation curve depicting the number of UMGS detected as a function of the number of metagenomic samples per continent. Data points represent the average of ten bootstrap replicates. The curve of best fit generated from an asymptotic regression is represented for each geographical region. In **a** and **c**, box lengths represent the IQR of the data, and the whiskers the lowest and highest values within 1.5 times the IQR from the first and third quartiles, respectively. Source Data

Subsequently, we correlated the prevalence and abundance of each UMGS and HGR genome with the geographical origin of the sample to infer any associations (Fig. 4). We investigated how many samples from the different continents each species was found at a relative abundance of more than 0.01% (Fig. 4a). In the majority of the sampled populations, the UMGS were less prevalent than the HGR genomes, a possible indication of why they have not been detected in previous genomic studies. However, the UMGS were more frequent, compared to the HGR genomes, among understudied samples from Africa and South America with non-Western lifestyles (Fig. 4a). This was particularly evident for a subset of 75 and 120 UMGS that were present at an abundance of more than 0.01% in more than 20% of the samples from Africa and South America, respectively (Fig. 4b). This was only the case for 6 and 16 HGR genomes, respectively, suggesting that some of our newly identified UMGS better represent the gut diversity present in the small number of samples from these two underrepresented populations.

To further evaluate the improvements provided by the UMGS for classification of the full metagenomic datasets, we assessed the percentage of reads that we were able to assign to HR, RefSeq and our UMGS dataset. With all the available genomes (HR, RefSeq, plus all UMGS), we observed a median classification of 72.8% (IQR = 65–81.1%). This represents an improvement of 23% over the use of a database comprising just HR, and of 17% over a combined set with HR and RefSeq. As the UMGS collection comprises over three times the number of gut species present in the HR database, this modest increase again suggests that the majority of these uncultured organisms are present at a lower abundance in most samples, compared to the gut isolate genomes.

After partitioning the data according to geographical origin, the small number of datasets from Africa (*n* = 21) and South America (*n* = 36) saw an improvement in read assignment of 215% and 278%, respectively (Fig. 4c). This confirms that some UMGS are much more abundant in these specific gut communities. In order to deduce how much diversity might remain undetected, we built an accumulation curve based on the number of UMGS retrieved as a function of the number of samples obtained from each continent (Fig. 4d). European and North American populations showed the greatest coverage, trending towards a saturation point. Conversely, in samples outside North America and Europe, new uncultured species are still detected at a consistent rate. These results underscore the importance of sampling underrepresented regions to continue to uncover the global diversity of the human gut microbiota.

## A distinctive functional repertoire

**Extended Data Fig. 8:**
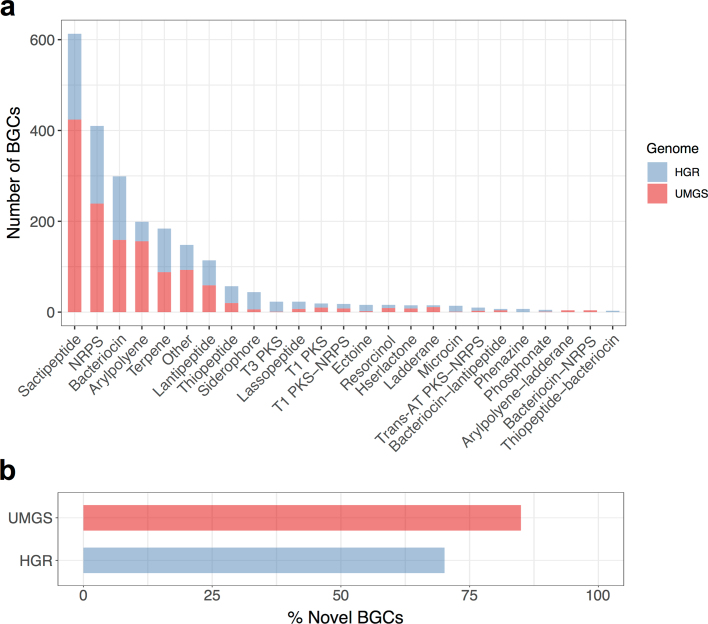
Biosynthetic gene clusters found in the human gut species. **a**, Number of BGCs found in the UMGS and the HGR genomes, subdivided by functional category. Only the 25 most abundant categories are depicted. PKS, polyketide synthases. **b**, Fraction of all BGCs that did not match the MIBiG database.

With access to 2,505 human gut species (1,952 UMGS and 553 HGR), we performed a comprehensive and in-depth functional characterization of the collective gut bacterial population. Using antiSMASH^35^, we screened for the presence of secondary metabolite biosynthetic gene clusters (BGCs) encoded within both the UMGS and HGR (Supplementary Table 5). We detected over 200 BGCs coding for sactipeptides, nonribosomal peptide synthetases (NRPSs) and bacteriocins (Extended Data Fig. 8a). Notably, 85% and 70% of the total BGCs detected in the UMGS and the HGR, respectively, represented novel clusters (that is, without a positive match in the Minimum Information about a Biosynthetic Gene (MIBiG) cluster database; Extended Data Fig. 8b). This suggests the potential presence of many undiscovered natural compounds produced by the intestinal microbiota with possible antimicrobial and/or biotechnological applications for future study.

**Fig. 5:**
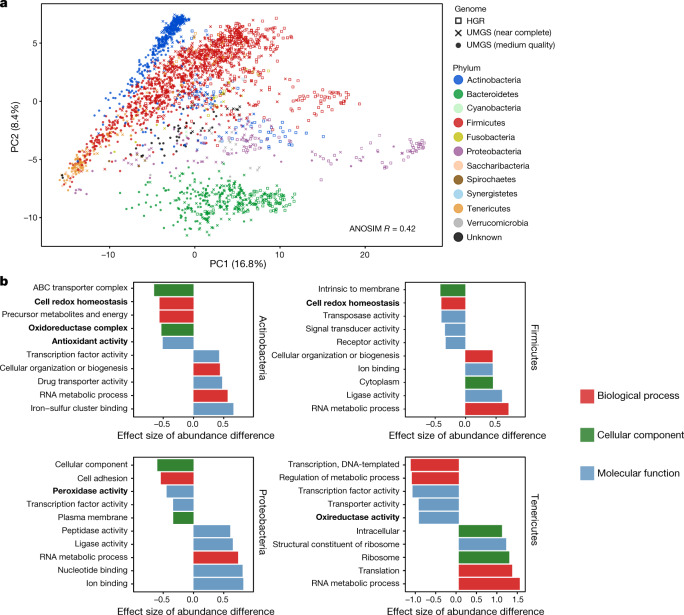
The uncultured species have a distinct functional capacity. **a**, Principal component analysis (PCA) based on GPs of the HGR (*n* = 553 genomes) and the UMGS (*n* = 1,952 genomes) coloured by phylum. **b**, GO functions differentially abundant between the HGR and UMGS genomes from Actinobacteria, Firmicutes, Proteobacteria and Tenericutes. The five functions with the highest and lowest effect size of abundance difference with a false discovery rate (FDR) <5% are represented. A positive effect size denotes overrepresentation in the UMGS genomes. GO terms related to redox functions are highlighted in bold.

**Extended Data Fig. 9:**
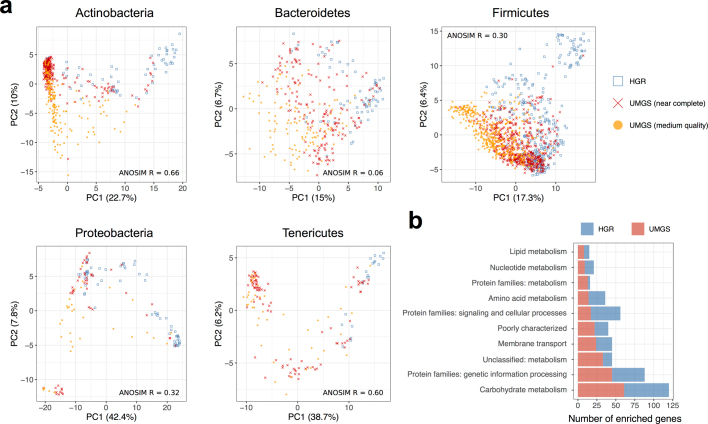
Functional capacity of cultured and uncultured species. **a**, PCA based on GPs of the 553 HGR genomes and the 1,952 UMGS for the five most prevalent phyla (Actinobacteria, Bacteroidetes, Firmicutes, Proteobacteria and Tenericutes). **b**, Number of genes found to be enriched with an absolute effect size >0.2 in either the UMGS or HGR genomes across the analyses of each of the five major phyla, grouped by their corresponding KEGG functional category.

We next applied complementary approaches to identify the most distinguishing traits between the UMGS and HGR genomes. First, from the predicted protein-coding sequences, we used InterProScan^36^ to generate annotations that were translated to 1,199 Genome Properties^37,38^ (GPs) and 115 metagenomics Gene Ontology^39,40^ (GO) slim terms—a summarized classification of GO annotations from metagenomic data^41^. Each GP—a functional attribute predicted to be encoded in a genome—was determined to be present, partially present or absent, depending on the number of proteins that were detected to be involved in that property. In parallel, we used GhostKOALA^42^ to generate KEGG Orthology (KO) annotations to track the differential abundance of specific functional categories across the UMGS and HGR sets. Globally, by analysing the repertoire of GPs according to the taxonomic composition, we observed a good separation by phylum (ANOSIM *R* = 0.42, *P* < 0.001), with the Bacteroidetes and Proteobacteria taxa in particular displaying very distinctive functional profiles (Fig. 5a). We further investigated the separation between the UMGS and HGR genomes within each phylum, which revealed a strong differentiation among Actinobacteria, Firmicutes, Proteobacteria and Tenericutes (ANOSIM *R* ≥ 0.30, Extended Data Fig. 9a). In particular, we detected 182, 207, 115 and 68 GPs particularly enriched in the UMGS genomes from Actinobacteria, Firmicutes, Proteobacteria and Tenericutes, respectively (*χ*^2^ test, adjusted *P* < 0.05), with only eight functions enriched within the Bacteroidetes group. Properties involved in iron metabolism and transport were among the 21 functions consistently enriched in the UMGS across these four most distinctive phyla (Extended Data Table 1).

Subsequently, by assessing the frequency of the GO and KO annotations, we were able to apply a quantitative approach to compare the HGR and UMGS functional repertoires. In general, KEGG pathways involved in carbohydrate metabolism were the most differentially abundant between the UMGS and HGR genomes, indicating distinct metabolic affinities between the cultured and uncultured species (Extended Data Fig. 9b). In the case of GO terms, less abundant genes (Wilcoxon rank-sum test, adjusted *P* < 0.05) within the UMGS were particularly associated with antioxidant and redox functions (Fig. 5b), indicative of lower tolerance to reactive oxygen species. If the UMGS correspond to strict anaerobes more sensitive to ambient oxygen, they are likely to be more difficult to isolate and culture. Conversely, in accordance with the GP results, we also observed an enrichment of genes coding for iron–sulfur and ion binding among the UMGS genomes, in addition to a variety of other functions. In anoxic conditions, the ferrous form of iron (Fe^2+^) that favours both sulfur and nitrogen ligands is most abundant^43^. An enrichment of iron–sulfur binding genes again suggests the UMGS may be better adapted to specific niches of the gastrointestinal tract with particularly low oxygen tension or high iron concentration, both of which generate high levels of ferrous ions in their environment^43^. Overall, these data show that the uncultured species described here carry specific functions that could explain their elusive nature, while raising awareness of biological traits underrepresented in current reference genome collections derived from pure bacterial cultures.

## Discussion

The human gut microbiota is one of the most studied microbial environments, but technical and practical constraints hinder our ability to isolate and sequence every constituent species. Metagenomic methods provide access to the uncultured microbial diversity, and here we have used these approaches to uncover 1,952 uncultured candidate bacterial species. Almost half of these putative species could not be classified at the genus level, suggesting that a substantial degree of bacterial diversity remains uncultured. This resource further expands and complements a recent study investigating the unexplored diversity of body-wide human microbiomes^44^.

As a result of our work, we now have representative genomes of 92,143 MAGs reconstructed from human gut assemblies and are able to classify 73% of the underlying read data. Nevertheless, both culturing and de novo analysis methods are inherently biased towards the most abundant organisms, meaning consistently less abundant species may still be missed. Furthermore, geographical regions such as Africa and South America are severely underrepresented in current studies. Therefore, expanding this analysis to large cohorts worldwide will be imperative for obtaining a complete overview of the human intestinal microbiota landscape. In addition, our work focused mainly on the study of bacterial genomes owing to the availability of more comprehensive reference databases and well-established standards and tools. However, as also shown here, metagenome assemblies generated from the gut microbiota include a wide range of other organisms such as archaea, eukaryotes and viruses that warrant a more thorough investigation.

Having access to comprehensive collections of bacterial genomes provides the ability to perform precise and computationally efficient reference-based genome analysis to achieve a detailed classification of microbial ecosystem composition. Our research is aimed at generating high-quality reference genomes, from pure cultures to MAGs, which will serve as a blueprint for metagenomic analysis of the human microbiota. The ability to leverage almost 2,000 additional species in future association and mechanistic studies will bring unprecedented power to investigate the impact of the microbiota in human health and disease.

## Methods

No statistical methods were used to predetermine sample size. The experiments were not randomized. The investigators were not blinded to allocation during experiments and outcome assessment.

### Metagenomic datasets

We extracted 13,133 sequencing runs classified as human gut metagenomes in the European Nucleotide Archive (ENA), encompassing 75 different studies (Supplementary Table 1). Metadata (location, age, health status and antibiotic usage) for each individual sampled was retrieved through the ENA API with the mg-toolkit (https://pypi.org/project/mg-toolkit/) and further curated by inspecting the publications linked to each project when available. Samples were classified as having been obtained from healthy individuals only if explicitly stated in their original study.

### De novo assembly and binning

Raw reads from each run were first assembled with SPAdes v.3.10.0^20^ with option *--*meta^21^. Thereafter, MetaBAT 2^15^ (v.2.12.1) was used to bin the assemblies using a minimum contig length threshold of 2,000 bp (option *--*minContig 2000) and default parameters. Depth of coverage required for the binning was inferred by mapping the raw reads back to their assemblies with BWA-MEM v.0.7.16^45^ and then calculating the corresponding read depths of each individual contig with samtools v.1.5^46^ (‘samtools view -Sbu’ followed by ‘samtools sort’) together with the jgi_summarize_bam_contig_depths function from MetaBAT 2. The QS of each metagenome-assembled genome (MAG) was estimated with CheckM v.1.0.7^22^ using the lineage_wf workflow and calculated as: level of completeness − 5 × contamination. Ribosomal RNAs (rRNAs) were detected with the cmsearch function from INFERNAL v.1.1.2^47^ (options -Z 1000 --hmmonly --cut_ga) using the Rfam^48^ covariance models of the bacterial 5S, 16S and 23S rRNAs. Total alignment length was inferred by the sum of all non-overlapping hits. Each gene was considered present if more than 80% of the expected sequence length was contained in the MAG. Transfer RNAs (tRNAs) were identified with tRNAscan-s.e. v.2.0^49^ using the bacterial tRNA model (option -B) and default parameters. Classification into high- and medium-quality MAGs was based on the criteria defined by the minimum information about a metagenome-assembled genome (MIMAG) standards^23^ (high: >90% completeness and <5% contamination, presence of 5S, 16S and 23S rRNA genes, and at least 18 tRNAs; medium: ≥ 50% completeness and <10% contamination). Given that only 240 of the MAGs with >90% completeness and <5% contamination passed the MIMAG thresholds regarding the presence of rRNA and tRNA genes due to known issues relating to the assembly of rRNA regions^16,50^, we refer to our highest quality MAGs as ‘near complete’^16^ instead. VirFinder v.1.1^51^ was used to predict the presence of viral contigs within the 13,133 human gut assemblies generated with SPAdes. This tool uses a *k*-mer-based, machine-learning approach to detect distinguishing signatures between virus and host (prokaryotic) sequences. Expected *P* values for the presence of viral sequences were calculated for each contig with ≥5 kb length and subsequently corrected for multiple testing using the Benjamini–Hochberg method with a FDR threshold of 10%.

### Assignment of MAGs to reference databases

Four reference databases were used to classify the set of MAGs recovered from the human gut assemblies: HR, RefSeq, GenBank and a collection of MAGs from public datasets. HR comprised a total of 2,468 high-quality genomes (>90% completeness, <5% contamination) retrieved from both the HMP catalogue (https://www.hmpdacc.org/catalog/) and the HGG^8^. From the RefSeq database, we used all the complete bacterial genomes available (*n* = 8,778) as of January 2018. In the case of GenBank, a total of 153,359 bacterial and 4,053 eukaryotic genomes (3,456 fungal and 597 protozoan genomes) deposited as of August 2018 were considered. Lastly, we surveyed 18,227 MAGs from the largest datasets publicly available as of August 2018^13,16–19^, including those deposited in the Integrated Microbial Genomes and Microbiomes (IMG/M) database^52^. For each database, the function ‘mash sketch’ from Mash v.2.0^53^ was used to convert the reference genomes into a MinHash sketch with default *k*-mer and sketch sizes. Then, the Mash distance between each MAG and the set of references was calculated with ‘mash dist’ to find the best match (that is, the reference genome with the lowest Mash distance). Subsequently, each MAG and its closest relative were aligned with dnadiff v.1.3 from MUMmer 3.23^54^ to compare each pair of genomes with regard to the fraction of the MAG aligned (aligned query, AQ) and ANI.

### Genome dereplication

To dereplicate the collection of unclassified bacterial MAGs (AQ <60% or ANI <95% against the target references), high-level similarity clusters were first generated with Mash^53^. In brief, a MinHash sketch was created for these genomes to perform an all-against-all comparison. Then, a hierarchical clustering was built from the Mash distance relationships and individual clusters were defined at a cut-off of 0.2. Each cluster was subsequently dereplicated with dRep v.2.2.2^55^ to extract the MAGs displaying the best quality and representing individual metagenomic species (MGS). dRep was run with options -pa 0.9 (primary cluster at 90%), -sa 0.95 (secondary cluster at 95%), -cm larger (coverage method: larger), -con 5 (contamination threshold of 5%). For the near-complete MAGs, the -nc parameter was set to 0.60 (coverage threshold of 60%), whereas for the medium-quality MAGs with a QS >50 this was changed to 0.30 (coverage threshold of 30%). The 2,468 HR genomes were also dereplicated into 956 representative species with dRep, using the criteria defined above for the near-complete MAGs. These included 553 species collected specifically from the human gut, referred to as HGR.

### Phylogenetic and taxonomic analyses

Genes were predicted using prodigal v.2.6.3^56^ (default single mode) and 40 universal core marker genes from each genome were extracted using specI v.1.0^32^. Phylogenetic trees were built by concatenating and aligning the marker genes with MUSCLE v.3.8.31. Marker genes absent only from specific genomes were kept in the alignment as missing data. Maximum-likelihood trees were constructed using RAxML v.8.1.15^57^ with option -m PROTGAMMAAUTO. All phylogenetic trees were visualized in iTOL^58^. Phylogenetic diversity was quantified by the sum of branch lengths using the phytools R package^59^.

Taxonomic classification of each MGS was performed with both CheckM and UniProtKB^29^. First, the function tree_qa from CheckM was used to infer the approximate phylogenetic placement of the MGS genome within the CheckM internal reference tree (which comprised 2,052 finished and 3,604 draft genomes). Those classified at least at the class rank were then compared with the taxonomic assignment deduced from protein alignments against UniProtKB (release 2018_04) using the blastp function of DIAMOND v.0.9.17.118^60^. A positive hit at the species level was inferred if ≥60% of the proteins had ≥80% of the sequence aligned with an amino acid identity of ≥96%, based on previously reported thresholds^26,33^. Genomes within UniProtKB were presumed to represent cultured species if labelled with a full species name lacking any of the following terms: uncultured, sp. or bacterium. For those MGS without an assigned species (UMGS), a genus-level boundary was set with the following criteria, as previously defined^61^: at least 50% of the proteins with an *e* value less than 1 × 10^−5^, a sequence identity of more than 40% and a query coverage above 50%. In case the taxon predicted with UniProt was missing from the CheckM reference database, the full lineage was manually inspected to determine the most likely annotation. Owing to possible mislabelling of the UniProt entries, the CheckM taxonomic lineage was kept if there were incongruences between both classifications. Lastly, the positioning of the UMGS genomes within the HGR phylogenetic tree was used to resolve further inconsistencies or misclassifications.

### Technical reproducibility and cluster quality

A random subset of 1,000 metagenomes (Supplementary Table 1) was tested with two additional approaches to assess the reproducibility of the MAGs generated here. With one of the methods, metagenomes were assembled with MEGAHIT v.1.1.3^24^ and subsequently binned with MetaBAT 2, MetaBAT 1 and MaxBin v.2.2.4^62^. A refinement step was then performed using the bin_refinement module from MetaWRAP v.1.0^25^ to combine and improve the results generated by the three binners. The second method involved a modified co-assembly approach, in which individual assemblies from the same study were first merged and dereplicated with CD-HIT v.4.7^63^ (cd-hit-est with option -c 0.99 defining a sequence identity threshold of 99%). Metagenomic datasets were then mapped to their merged, non-redundant assembly with BWA-MEM to obtain co-abundance information for binning with MetaBAT 2 (with option --minContig 2000). The resulting MAGs with a QS >50 obtained with each method were compared to the MAGs recovered with our main pipeline (individual assembly with SPAdes, plus binning with MetaBAT 2) for the same 1,000 datasets, using the combined Mash and MUMmer workflow described above.

To further assess the level of potential contamination of the MGS reported, we analysed the quality of the Mash clusters containing each MGS using the Matthews Correlation Coefficient (MCC). First, CompareM v.0.0.23 (https://github.com/dparks1134/CompareM) was used to analyse the average amino acid identity (AAI) of the specI marker genes within and between Mash clusters. To be able to estimate the MCCs, true positives, false negatives, false positives and true negatives were determined based on three different AAI thresholds: 90%, 95% and 97%. For each pairwise comparison, we considered a true positive when both MAGs belonged to the same cluster and had an AAI equal to or above the threshold; false negatives if they belonged to the same cluster, but the AAI was below the threshold; false positives when the genomes were included in different clusters, but their AAI was equal to or above the threshold; and true negatives corresponded to genomes from different clusters with an AAI below the threshold. Thereafter, MCCs were calculated with the mcc function from the mltools^64^ R package. Possible values range from −1 to 1, with 1 indicating perfect agreement between the Mash clustering and the marker genes AAI.

### Functional characterization

Functional prediction analyses were carried out for the 1,952 UMGS and the dereplicated set of 553 HGR genomes. Predicted genes were first functionally characterized with InterProScan v.5.27-66.0^36^ with options -goterms and -pa. The presence of microbial BGCs was inferred with antiSMASH 4^35^, using option --knowclusterblast to determine the number of BGCs that matched the MIBiG repository. GO^39,40^ annotations were deduced for each gene based on the InterPro (IPR) entries, and translated to GPs^37,38^ using the assign_genome_properties.pl script present in http://github.com/ebi-pf-team/genome-properties. GhostKOALA^42^ was used to generate KO annotations of the protein-coding sequences. Differential abundance analysis of GO slim and KO term frequencies between the UMGS and HGR genomes was performed with the compositional data analysis tool ALDEx2^65^. Because we were evaluating genomes with differing lengths and degrees of completeness, this method was used to take into account discrepancies in total gene counts. The aldex.clr function was used with 128 Monte Carlo instances sampled from a Dirichlet distribution to generate a distribution of probabilities for each GO slim/KO term consistent with the observed data. These were subsequently converted to distributions of log ratios to account for the compositional nature of the data. The aldex.effect function was used to calculate the expected value of the difference between distributions of each group (median log_2_ difference), the expected value of the pooled group variance (median log_2_ dispersion) and the standardized effect sizes on the abundance difference of each GO/KO classification. The effect-size measure used is similar in concept to Cohen’s *d* but is calculated on the distributions themselves rather than on the summary statistics of those distributions, resulting in metrics that are relatively robust and efficient^66^. Lastly, the aldex.ttest was used to perform non-parametric Wilcoxon rank-sum tests on the GO/KO frequencies between the two test groups (UMGS and HGR). GPs, classified as ‘yes’, ‘no’ and ‘partial’ were converted to 2, 0 and 1, respectively, and those more prevalent specifically among the UMGS genomes were detected with a two-tailed *χ*^2^ test. The expected *P* values from all the statistical tests were corrected for multiple testing with the Benjamini–Hochberg method. A PCA was carried out on the GP distributions of the HGR and UMGS genomes, using the FactorMineR^67^ package. Separation according to phylum and genome type was assessed with the ANOSIM test based on the Gower distances between the GP profiles.

### Species prevalence and abundance

Read classification of the 13,133 human gut metagenomic datasets was performed with sourmash v.2.0.0a4^68^ against the HR, RefSeq and UMGS genome collections. Signature files were generated for both the reference (FASTA) and query (FASTQ) files, with ‘sourmash compute --scaled 1000 -k 31 --track-abundance’. For each set of references, a lowest common ancestor database was created (‘sourmash lca index --scaled 1000 -k 31’), with each genome representing a unique species lineage. Raw reads were then compared with ‘sourmash lca gather’ against each database. Species prevalence and abundance was determined with BWA-MEM, where species presence was inferred by assessing the level of genome coverage, mean read depth and depth evenness. First, we calculated depth and variation penalty scores corresponding to the missing coverage (100% − genome coverage) multiplied by either the log(mean depth) or the depth coefficient of variation (defined as the standard deviation of read depth divided by the mean), respectively. These metrics allowed us to gauge both coverage and depth simultaneously, as genomes that have a high mean depth (or high depth variation) but are not well covered are less likely to be present in the sample than those that have the same level of coverage with lower read depth. Thresholds for determining genome presence were set at a minimum coverage of at least 60%, and both depth and variation penalty scores at a maximum of the 99th percentile (Extended Data Fig. 7). Relative abundance of each species was determined by the proportion of uniquely mapped and correctly paired reads (filtered using ‘samtools view -q 1 -f 2’) out of the total read count. Accumulation curves based on the number of UMGS detected per geographical region were bootstrapped ten times at each sampling interval. Asymptotic regressions were performed using the SSasymp and nls functions from the R stats package^69^.

### Reporting summary

Further information on research design is available in the Nature Research Reporting Summary linked to this paper.

### Code availability

Custom scripts used to generate data and figures are available at https://github.com/Finn-Lab/MGS-gut.

## Online content

Any methods, additional references, Nature Research reporting summaries, source data, statements of data availability and associated accession codes are available at 10.1038/s41586-019-0965-1.

### Supplementary information

Supplementary Discussion Technical details and results concerning the reproducibility of the assembly/binning pipeline; detection of non-prokaryotic bins; genome dereplication and quality assessment, and comparison with publicly available uncultured genomes.

Reporting Summary

Supplementary Table 1 Information on the 13,133 human gut datasets analysed.

Supplementary Table 2 Genome bins predicted to belong to eukaryotic organisms.

Supplementary Table 3 Information on the 39,891 near-complete bacterial MAGs generated in this work.

Supplementary Table 4 Detailed genome and quality statistics of the 1,952 UMGS identified in this work.

Supplementary Table 5 Number and type of biosynthetic gene clusters detected with antiSMASH in the 1,952 UMGS.

### Source data

Source Data Fig. 4

Source Data Extended Data Fig. 2

Source Data Extended Data Fig. 5

## Figures and Tables

**Extended Data Table 1:**
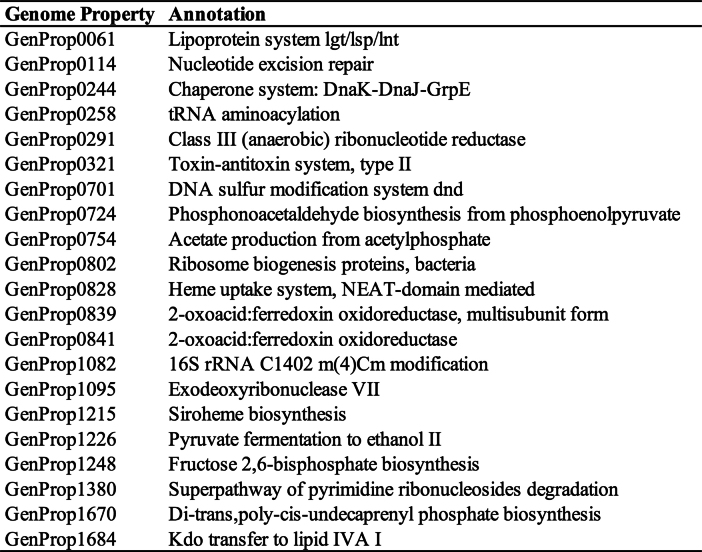
Genome Properties overrepresented in the UMGS genomes

Genome Properties found to be overrepresented among the UMGS compared to the HGR from Actinobacteria, Firmicutes, Proteobacteria and Tenericutes. Statistical significance was assessed with a two-tailed *χ*^2^ test on the proportion of functions with ‘partial’ and ‘yes’ in relation to the total counts of all the functions detected per genome type.

## Data Availability

The UMGS genomes generated in this work were deposited in ENA, under the study accession ERP108418. The 92,143 MAGs with QS >50, as well as the quantification results from BWA and sourmash, all phylogenetic trees and the functional analysis results with InterProScan, GP and GhostKOALA are available at ftp://ftp.ebi.ac.uk/pub/databases/metagenomics/umgs_analyses/.
