# Supplementary material for: A new genomic blueprint of the human gut microbiota
Source: Nature. 2019 Feb 11;568(7753):499–504. doi: 10.1038/s41586-019-0965-1 (PMC6784870; doi:10.1038/s41586-019-0965-1)
Supplement: Supplementary file 1 — Technical details and results concerning the reproducibility of the assembly/binning pipeline; detection of non-prokaryotic bins; genome dereplication and quality assessment, and comparison with publicly available uncultured genomes. [file 41586_2019_965_MOESM1_ESM.pdf]

nature

SUPPLEMENTARY INFORMATION

https://doi.org/10.1038/s41586-019-0965-1

In the format provided by the authors and unedited.

A new genomic blueprint of the human gut microbiota

Alexandre Almeida[1,2*], Alex L. Mitchell[1], Miguel Boland[1], Samuel C. Forster[2,3,4], Gregory B. Gloor[5], Aleksandra Tarkowska[1], Trevor D. Lawley[2] & Robert D. Finn[1*]

[1]European Bioinformatics Institute (EMBL-EBI), Wellcome Genome Campus, Hinxton, UK. [2]Wellcome Sanger Institute, Wellcome Genome Campus, Hinxton, UK. [3]Centre for Innate Immunity and Infectious Diseases, Hudson Institute of Medical Research, Clayton, Victoria, Australia. [4]Department of Molecular and Translational Sciences, Monash University, Clayton, Victoria, Australia. [5]Department of Biochemistry, University of Western Ontario, London, Ontario, Canada. *e-mail: aalmeida@ebi.ac.uk; rdf@ebi.ac.uk

Supplementary Discussion

Technical reproducibility of the assembly and binning pipeline

To assess the robustness of the reported metagenome-assembled genomes (MAGs) in relation to our assembly/binning methodology, we re-analysed a random subset of 1,000 metagenome datasets (Supplementary Table 1) with two independent approaches. One relied on using the MEGAHIT[24] assembler together with the results from three binning tools for further refinement with the MetaWRAP[25] pipeline. The other method involved a modified co-assembly approach, where multiple samples from the same study were mapped to a common merged assembly for subsequent binning with MetaBAT[15]. The resulting bins from these two pipelines with quality score (QS) > 50 were compared with the original MAGs generated with the same 1,000 datasets. More than 98% of the MAGs generated with both approaches matched the original set (Extended Data Fig. 3), suggesting that the MAGs here described are highly reproducible and largely independent of the method used for assembly and binning.

Detecting non-prokaryotic bins

Although we mainly focused on the bacterial diversity present in the recovered MAGs, we investigated further how many of our bins represented known eukaryotes or viral sequences that form part of the human gut microbiota. We compared all bins not assigned to either bacteria or archaea by CheckM (n = 39,967) against the GenBank collection of all fungal and protozoan genomes. A total of 857 bins had at least 60% of their genome aligned to a known eukaryotic organism (854 to protozoa and 3 to fungi; Supplementary Table 2). As viral sequences were rarely found to be binned together and instead contained among other prokaryotic or eukaryotic sequences, we screened the original metagenome assemblies for the presence of viral contigs. Using VirFinder[51], we detected 6,555 viral contigs with ≥ 5 kb length (false discovery rate, FDR < 10%) among 1,615 human gut assemblies.

Genome de-replication and quality assessment

Focusing on the 11,888 near-complete bacterial MAGs that were not assigned to HR or RefSeq (Fig. 1b), we performed a two-step de-replication process. First, we clustered the 11,888 unclassified MAGs into a set of 702 similarity groups using a Mash[53] distance of 0.2 (i.e. approximate average nucleotide identity, ANI ≥ 80%). To identify potential biases in the underlying sequence data — for example, specific laboratory and/or batch effects — the distribution of MAGs extracted from different samples and studies per cluster was examined (Extended Data Fig. 5). There was a strong correlation between the number of MAGs per similarity group and the corresponding number of samples (R^2 = 0.94) and studies (R^2 = 0.89) from which they were obtained, suggesting that recurrent MAGs were the result of multiple, independent observations. Thereafter, we de-replicated the MAGs included within each Mash cluster by extracting the best quality representative genomes. For the de-replication process, MAGs were defined as belonging to the same species using previously-defined boundaries for species demarcation (> 95% ANI over an alignment fraction of at least 60%)[26,27].

After de-replication, we controlled for the presence of contaminated/chimeric metagenomic species (MGS) by checking the average amino acid identity (AAI) of universal marker genes recovered with specI[32] from each MGS in their respective Mash clusters (Extended Data Fig. 6c). We calculated the Matthews Correlation Coefficient (MCC) for the pairwise comparisons of MAGs within and between each cluster using an AAI cut-off of 97%. The 2,068 near-complete and medium-quality MGS presented a median MCC of 1.00 (interquartile range, IQR = 0.91–1.00), showing that the identity of the marker genes between MAGs is largely in line with the Mash clustering structure. Although special care should always be taken when analysing MAGs — especially genomic regions lacking universal

marker genes, such as mobile genetic elements — these results reinforce the notion that most of our recovered MGS present very low levels of contamination.

Comparison with publicly available uncultured genomes

With the increasing number of genomes and metagenomes available from a wide-range of environments, we assessed how many of our unclassified metagenomic species (UMGS) matched publicly available databases that included other uncultured genomes. In particular, we surveyed all 153,359 genomes from GenBank, together with the largest MAG datasets available as of August 2018[13,16–19] including those deposited in the Integrated Microbial Genomes and Microbiomes (IMG/M) database[52]. A total of 1,445 UMGS (74%) did not match any of these datasets (using our genome-based species criteria), showing that the majority of the UMGS consist of completely novel genomes (Supplementary Table 4).
