# Supplementary material for: A new genomic blueprint of the human gut microbiota
Source: Nature. 2019 Feb 11;568(7753):499–504. doi: 10.1038/s41586-019-0965-1 (PMC6784870; doi:10.1038/s41586-019-0965-1)
Supplement: Supplementary file 2 — Reporting Summary [file 41586_2019_965_MOESM2_ESM.pdf]

natureresearch

Corresponding author(s): Alexandre Almeida and Robert D. Finn

Last updated by author(s): Feb 28, 2019

Reporting Summary

Nature Research wishes to improve the reproducibility of the work that we publish. This form provides structure for consistency and transparency in reporting. For further information on Nature Research policies, see Authors & Referees and the Editorial Policy Checklist.

Statistics

For all statistical analyses, confirm that the following items are present in the figure legend, table legend, main text, or Methods section.

n/a	Confirmed	
☐	☒	The exact sample size (*n*) for each experimental group/condition, given as a discrete number and unit of measurement
☐	☒	A statement on whether measurements were taken from distinct samples or whether the same sample was measured repeatedly
☐	☒	The statistical test(s) used AND whether they are one- or two-sided<br>*Only common tests should be described solely by name; describe more complex techniques in the Methods section.*
☐	☒	A description of all covariates tested
☐	☒	A description of any assumptions or corrections, such as tests of normality and adjustment for multiple comparisons
☐	☒	A full description of the statistical parameters including central tendency (e.g. means) or other basic estimates (e.g. regression coefficient) AND variation (e.g. standard deviation) or associated estimates of uncertainty (e.g. confidence intervals)
☐	☒	For null hypothesis testing, the test statistic (e.g. *F*, *t*, *r*) with confidence intervals, effect sizes, degrees of freedom and *P* value noted<br>*Give P values as exact values whenever suitable.*
☐	☒	For Bayesian analysis, information on the choice of priors and Markov chain Monte Carlo settings
☒	☐	For hierarchical and complex designs, identification of the appropriate level for tests and full reporting of outcomes
☐	☒	Estimates of effect sizes (e.g. Cohen's *d*, Pearson's *r*), indicating how they were calculated

Our web collection on statistics for biologists contains articles on many of the points above.

Software and code

Policy information about availability of computer code

Data collection	mg-toolkit (https://pypi.org/project/mg-toolkit/); European Nucleotide Archive (https://www.ebi.ac.uk/ena)
Data analysis	R v3.4.1; Python v2.7.5 and v3.6.5; SPAdes v3.10.0; MetaBAT v2.12.1; BWA v0.7.16; samtools v1.5; CheckM v1.0.7; Mash v2.0; MUMmer v3.23; specI v1.0; MUSCLE v3.8.31; DIAMOND v0.9.17.118; prodigal v2.6.3; InterProScan v5.27-66.0; antiSMASH 4; ALDEx2; sourmash v2.0.0a4; phytools v0.6-44; GhostKOALA; VirFinder v1.1; CompareM v0.0.23; MEGAHIT v1.1.3; MetaWRAP v1.0; MaxBin v2.2.4; mltools v0.3.5; RAxML v8.1.15; CD-HIT v4.7; tRNAscan-SE v2.0; INFERNAL v1.1.2; dRep v2.2.2

For manuscripts utilizing custom algorithms or software that are central to the research but not yet described in published literature, software must be made available to editors/reviewers. We strongly encourage code deposition in a community repository (e.g. GitHub). See the Nature Research guidelines for submitting code & software for further information.

Data

Policy information about availability of data

All manuscripts must include a data availability statement. This statement should provide the following information, where applicable:
- Accession codes, unique identifiers, or web links for publicly available datasets
- A list of figures that have associated raw data
- A description of any restrictions on data availability

The UMGS genomes generated in this work were deposited in ENA, under the study accession ERP108418. The 92,143 MAGs with QS > 50, as well as the quantification results from BWA and sourmash, all phylogenetic trees and the functional analysis results with InterProScan, GP and GhostKOALA are available in the following public FTP: ftp://ftp.ebi.ac.uk/pub/databases/metagenomics/umgs_analyses/.

Field-specific reporting

Please select the one below that is the best fit for your research. If you are not sure, read the appropriate sections before making your selection.

☒ Life sciences ☐ Behavioural & social sciences ☐ Ecological, evolutionary & environmental sciences

For a reference copy of the document with all sections, see nature.com/documents/nr-reporting-summary-flat.pdf

Life sciences study design

All studies must disclose on these points even when the disclosure is negative.

Sample size	We analysed 13,133 human gut metagenomic datasets, corresponding to a large portion of the human gut metagenomic data publicly available. No sample size calculation was performed, as we were limited by the computational resources available. Our results showed that with the number of datasets we analysed the amount of uncultured species detected begins to plateau for North American and European populations, showing that this level of scale was sufficient to obtain a good coverage of the gut microbiota diversity of these regions.
Data exclusions	1,283 human gut datasets were excluded as they did not generate genome bins with MetaBAT 2.
Replication	We assessed the reproducibility of the method used for generating the metagenome-assembled genomes (MAGs) by re-analysing a subset of 1,000 random gut metagenomes with MetaWRAP and with a modified co-assembly approach. With both strategies, > 98% of the MAGs matched our original set, indicating that they are highly robust to the choice of assembly/binning method. In addition, the distribution of MAGs extracted from different samples and studies was examined. There was a strong correlation between the number of similar MAGs extracted and the number of corresponding samples and studies from which they were obtained, suggesting that recurrent MAGs were the result of multiple, independent observations.
Randomization	Randomization was not relevant to this study. We highlight the presence of geographical biases (towards North American and European populations) of the publicly available metagenomic datasets we analysed.
Blinding	Not relevant to this study, as we analysed publicly available metagenomic data.

Reporting for specific materials, systems and methods

We require information from authors about some types of materials, experimental systems and methods used in many studies. Here, indicate whether each material, system or method listed is relevant to your study. If you are not sure if a list item applies to your research, read the appropriate section before selecting a response.

Materials & experimental systems

n/a	Involved in the study
☒	☐ Antibodies
☒	☐ Eukaryotic cell lines
☒	☐ Palaeontology
☒	☐ Animals and other organisms
☒	☐ Human research participants
☒	☐ Clinical data

Methods

n/a	Involved in the study
☒	☐ ChIP-seq
☒	☐ Flow cytometry
☒	☐ MRI-based neuroimaging
